# A novel risk classification model integrating CEA, ctDNA, and pTN stage for stage 3 colon cancer: a post hoc analysis of the IDEA-France trial

**DOI:** 10.1093/oncolo/oyae140

**Published:** 2024-07-15

**Authors:** Thomas Samaille, Antoine Falcoz, Romain Cohen, Pierre Laurent-Puig, Thierry André, Julien Taieb, Edouard Auclin, Dewi Vernerey

**Affiliations:** Department of Medical Oncology, Saint-Antoine Hospital, Sorbonne Université, Paris, France; Methodology and Quality of Life Unit in Oncology, University Hospital of Besançon, Besançon, France; Department of Medical Oncology, Saint-Antoine Hospital, Sorbonne Université, Paris, France; Institut du cancer Paris CARPEM, Georges Pompidou European Hospital, AP-HP, Université Paris Cité, Paris, France; Department of Medical Oncology, Saint-Antoine Hospital, Sorbonne Université, Paris, France; Department of Gastroenterology and GI oncology, Georges Pompidou European Hospital, SIRIC CARPE, Université Paris-Cité, Paris, France; Department of Medical Oncology, Georges Pompidou European Hospital, AP-HP, Université Paris Cité, Paris, France; Methodology and Quality of Life Unit in Oncology, University Hospital of Besançon, Besançon, France

**Keywords:** carcinoembryonic antigen, ctDNA, colon cancer, TN staging

## Abstract

**Background:**

We assessed the added value of incorporating carcinoembryonic antigen (CEA) to circulating tumor DNA (ctDNA) and pathological TN (pTN) stage for risk classification in stage 3 colon cancer (CC).

**Patients and Methods:**

We retrospectively analyzed postoperative CEA values in patients with CC from the IDEA-France phase 3 trial. The relation between disease-free survival (DFS) and CEA was modeled through restricted cubic splines. Prognostic value of CEA, ctDNA, and pTN was assessed with the Kaplan-Meier method. Multivariate analysis was used to identify prognostic and predictive factors for DFS.

**Results:**

Among 696 patients (35%), CEA values were retrievable, and for 405 (20%) both CEA and ctDNA were available. An optimized CEA threshold of 2 ng/mL was identified, the 3-year DFS was 66.4% for patients above the threshold and 80.9% for those below (HR, 1.74; 95% CI, 1.33-2.28, *P* < .001). In multivariate analysis, CEA ≥ 2 ng/mL contributed significantly to model variability, becoming an independent prognostic factor for DFS (HR, 1.82; 95% CI,1.27-2.59), alongside ctDNA (HR, 1.88; 95% CI, 1.16-3.03) and pTN (HR, 1.78; 95% CI, 1.24-2.54). A novel integrated risk classification combining CEA, ctDNA, and pTN stage reclassified 19.8% of pT4/N2 patients as low risk and 2.5% of pT3/N1 patients as high risk. This new classification demonstrated the 3-year DFS of 80.8% for low-risk patients and 55.4% for high-risk patients (HR, 2.66, 95% CI, 1.84-3.86, *P* < .001).

**Conclusions:**

Postoperative CEA value is a prognostic factor for DFS in stage 3 CC, independently of ctDNA and pTN. It advocates for systematic reporting in future adjuvant trials. Integrating both biomarkers with pTN could refine risk classification in stage 3 CC.

## Teaser

This study assessed the added value of incorporating carcinoembryonic antigen to circulating tumor DNA and pathological TN stage for risk classification in stage 3 colon cancer.

**Implications for Practice**

The retrospective analysis of the IDEA-France trial emphasized the prognostic role of CEA for disease-free survival in stage 3 colon cancer. Post-operative CEA values between 2 and 5 ng/mL, often considered as normal, identified patients with an increased risk of relapse. This finding was confirmed in 2 external validation cohorts. CEA prognostic value was found independent of pathological TN and ctDNA. As CEA is routinely available, the integrated risk classification system introduced here can easily translate into clinical practice to improve prognostic performance and better tailor treatment strategies for patients with colorectal cancer.

## Introduction

Stage 3 colon cancer (CC) treatment primarily relies on surgery followed by adjuvant chemotherapy, incorporating fluoropyrimidine and oxaliplatin,^1-3^ with potential cumulative toxicities such as peripheral neuropathy. Despite adjuvant chemotherapy, recurrence still occurs in approximately 20% of low-risk stage 3 (pT1-3 and N1) and 40% of high-risk stage 3 (T4 and/or N2) cases.^4^ Recently, recommendations on optimal adjuvant chemotherapy duration were modified based on the results of the IDEA trial.^4^ This trial established a 3-month adjuvant treatment with capecitabine and oxaliplatin (CAPOX) for patients with pathological (p)T1-3 and N1 (pT1-3/N1) tumors, while favoring a 6-month treatment with FOLFOX or CAPOX for patients with pT4 and/or N2 (pT4/N2). These results established the pTN stage as a routinely used parameter for decision-making.

Circulating tumor DNA (ctDNA) emerged as a molecular biomarker recently proposed to aid therapeutic decision about adjuvant chemotherapy.^5,6^ Building on the concept of Molecular Residual Disease in hematology, it was suggested that the presence of ctDNA indicates persistent tumor cells that could be eliminated by chemotherapy. While this approach has been evaluated in stage 2 colorectal cancers, where chemotherapy might be avoided in patients without ctDNA,^7,8^ its clinical utility in stage 3 CC, with a higher relapse risk and indication for adjuvant therapy in all patients, remains to be proven.

Carcinoembryonic antigen (CEA) is a well-established, cost-effective biological tumor marker used in colorectal cancer since 1965.^9^ Its current use is restrained to helping the diagnosis of relapse in localized cancer and monitoring chemotherapy efficacy in the metastatic setting. A value above 5 ng/mL is typically considered as abnormal, although this may vary based on national guidelines and patient characteristics, such as smoking status. Recent studies have focused on optimizing the use of CEA, suggesting modified threshold may improve its prognostic and predictive value for adding oxaliplatin to fluoropyrimidine in stage 2 cancer^10^ and improving its prognostic value in stage 3 cancer.^11^ Its correlation with ctDNA positivity remains largely unknown.

Only a few studies to date have reported on combined analysis of pTN, ctDNA, and CEA for the prognosis of colorectal cancer.^8,12,13^ In surveillance strategies, a combination of CEA and imaging has been reported as efficient as ctDNA in detecting relapses.^13^ In a Danish and Spanish study evaluating ctDNA follow-up in 168 patients with stage 3 cancers, positive ctDNA was reported as the main prognostic factor. However, postoperative CEA remained an independent factor for recurrence-free survival in multivariate analyses.^12^

In this context, we performed a post hoc analysis of the IDEA-France phase 3 trial to (i) optimize the use of CEA as a prognostic factor for disease-free survival (DFS), (ii) evaluate the relative contributions of CEA, ctDNA, and pTN stage, (iii) propose a new integrated risk classification combining optimized CEA threshold, ctDNA status, and pTN stage, and (iv) assess the potential of this new risk classification to better identify patients with improved outcomes with longer adjuvant chemotherapy.

## Methods

### Study design and patients

The IDEA international collaboration aimed to assess the noninferiority of a 3-month duration compared to a 6-month duration of adjuvant oxaliplatin-based chemotherapy in stage 3 CC.^4,14^ In the IDEA-France phase 3 trial, a total of 2010 patients were included in 129 centers from May 2009 to May 2014. They were randomized (1:1) between 3 and 6 months of adjuvant chemotherapy with modified FOLFOX6 or CAPOX.^15^ In the IDEA-France cohort, approximately 90% of the randomized patients received FOLFOX6, while CAPOX was used in approximately 40% of patients included in the whole international collaboration. The final results of the IDEA trial on the prognostic value of ctDNA and DFS have been previously published.^6^

Briefly, included patients were aged ≥ 18, had an ECOG performance status of 0-2, were diagnosed with stage 3 histologically confirmed CC, defined as a tumor location >12 cm from the anal verge by endoscopy and/or above the peritoneal reflection at surgery (high rectum), who underwent curative surgery 8 weeks or less before randomization, and had postoperative CEA level below 10 ng/mL.

For external validation, the relationship between CEA values and DFS was also modeled using 2 independent cohorts. The first cohort was composed of 1292 patients from the MOSAIC phase 3 trial (NCT00275210), which showed a survival improvement with the addition of oxaliplatin to adjuvant fluoropyrimidine in patients with CC.^1^ The second cohort comprised 2480 patients from the PETACC-8 phase 3 trial (NCT00265811811), which failed to show a survival benefit for the adjuvant combination of FOLFOX and cetuximab in patients with stage 3 CC.^16^

### CEA and ctDNA assessments

Postoperative CEA values were not centralized and were retrospectively retrieved from the medical files of patients. The ctDNA analysis was centralized and formed part of an ancillary translational study of the IDEA-France trial. This analysis relied on the detection of *WIF1* and *NPY* gene hypermethylation by multiplex droplet-based digital PCR using the QX200 platform (Bio-Rad), as previously published on 1017 participants of the IDEA-France trial.^6,17^

### Statistical analysis

Median values (interquartile range) and frequencies (percentage) were provided for descriptive statistics of continuous and categorical variables, respectively. The Student’s *t* test and the chi-square test (or the Fisher’s exact test when appropriate) were used to compare between medians and proportions used, respectively. For continuous assessments, the association between CEA and DFS was investigated with the restricted cubic splines method with graphical evaluation.

DFS was defined as the time between randomization and the occurrence of local/distant relapse, second colorectal/rectal occurrence, or death, whichever happened first. Patients alive without relapse and second colorectal/rectal cancer were censored at the date of their last follow-up. DFS was assessed through the Kaplan-Meier method and described using median or rate at specific time points with corresponding 95% CI.

The association of factors with DFS was first assessed by the univariate Cox-proportional-hazards model, providing hazard ratios (HRs) and 95% CIs. A multivariate Cox-proportional-hazards model, integrating parameters of interest (ctDNA, CEA, TN stage, and treatment duration) was then proposed. The differential DFS treatment effect (3 vs 6 months) was evaluated with an interaction term in the Cox-regression model and illustrated with Kaplan-Meier curves.

All analyses were performed using SAS version 9.4 (SAS Institute, Cary NC) and R software version 4.1.1 (R Development Core Team, Vienna, Austria; http://www.r-project.org). *P*-values <.05 were considered statistically significant, provided for an exploratory purpose, as no correction for multiple testing was made; a threshold of 0.1 was used for interaction terms. All tests were 2-sided.

## Results

### Patient characteristics

Out of 2010 patients included in the IDEA-France trial, CEA values were retrieved for 696 (35%; Supplementary Figure S1), showing similar characteristics and outcomes (Supplementary Table S1 and Figure S2). Both CEA and ctDNA status were available for 405 patients (20%). The baseline characteristics of these 405 patients, included in all analyses, and a comparison to the whole population are shown in Table 1.

**Table 1.** Characteristics of the whole study population and patients included and excluded from analysis.

	mITT population		Population with CEA and ctDNA		Population without CEA and/or ctDNA	
	*N* = 2010		*n* = 405		*n* = 1605	
	*n*	%	*n*	%	*n*	%
Age, years						
Mean (SD)	63.9 (9.4)		62.5 (9.9)		64.3 (9.2)	
Median (Q1-Q3)	64.7 (58.1-70.8)		63.5 (56.4-69.6)		65.2 (58.5-71.1)	
≤70	1443	71.8	310	76.5	1133	70.6
>70	567	28.2	95	23.5	472	29.4
Sex						
Male	1144	56.9	218	53.8	926	57.7
Female	866	43.1	187	46.2	679	42.3
ECOG PS						
0	1479	73.6	325	80.3	1154	71.9
1-2	531	26.4	80	19.7	451	28.1
Tumor and node stage						
T1-3 and N1	1246	62.0	249	61.5	997	62.2
T4 and/or N2	764	38.0	156	38.5	608	37.8
Chemotherapy duration						
3 months	1002	49.9	202	49.9	800	49.8
FOLFOX	895	89.3	178	88.1	717	89.6
CAPOX	107	10.7	24	11.9	83	10.4
6 months	1008	50.1	203	50.1	805	50.2
FOLFOX	914	90.7	181	89.2	733	91.1
CAPOX	94	9.3	22	10.8	72	8.9
Primary tumor site						
Left	1161	60.4	251	62.3	910	59.8
Right	746	38.8	148	36.7	598	39.4
Both	16	0.8	4	1.0	12	0.8
Missing	87	—	2	—	85	—
Histologic grade						
Low grade	1764	91.7	345	89.4	1419	92.3
High grade	159	8.3	41	10.6	118	7.7
Missing	87	—	19	—	68	—

Abbreviations: mIIT, modified intent-to-treat population; CEA, carcinoembryonic antigen; ctDNA, circulating DNA; ECOG PS, Eastern Cooperative Oncology Group Performance Status.

### Association between CEA and DFS

**Figure 1.**
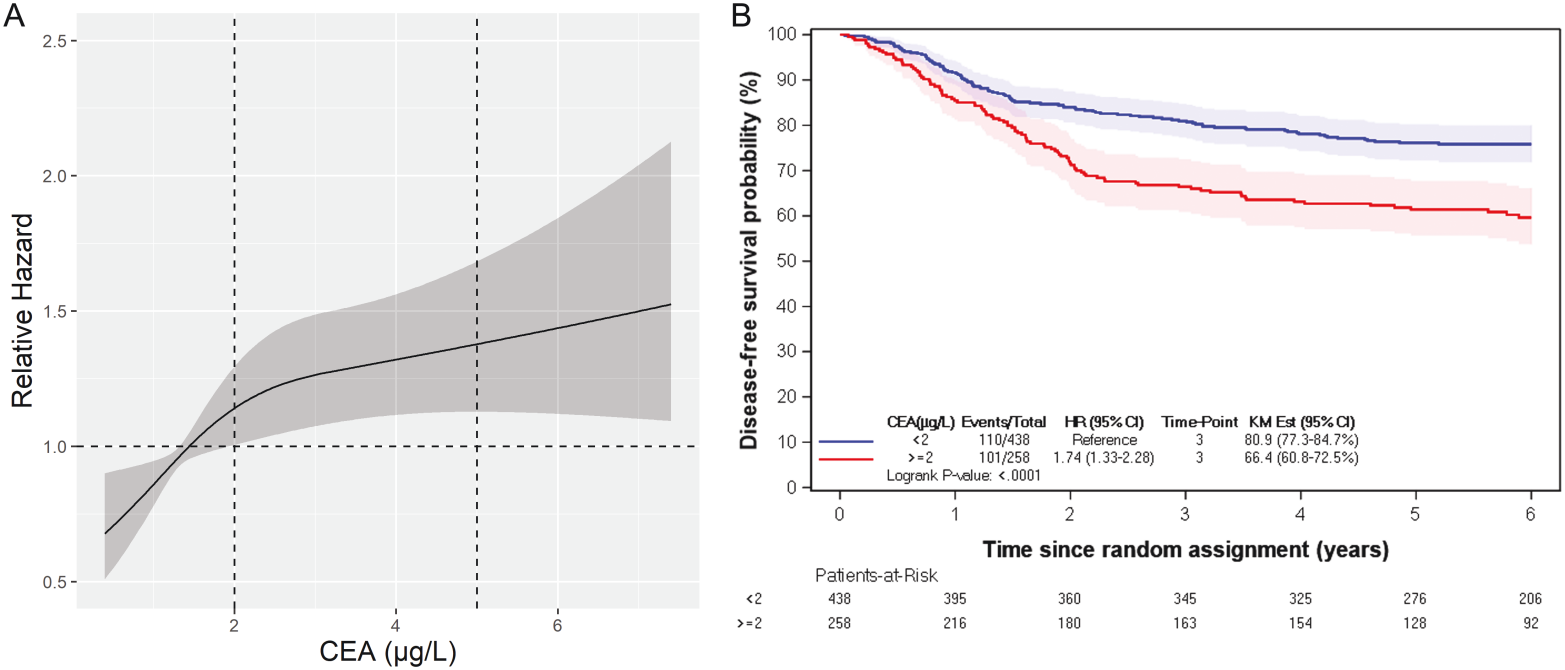
Association between CEA value and DFS by the restricted cubic spline method (A). Kaplan-Meier estimates of DFS according to the optimized CEA threshold of 2 ng/mL (B) in the IDEA trial. Abbreviations: CEA, carcinoembryonic antigen; DFS, disease-free survival.

The restricted cubic spline analysis showed a continuous square-root relationship between DFS and CEA level (Figure 1A). In the IDEA-France population with available CEA value (*n* = 696), a threshold set at 2 ng/mL identified a subgroup at significantly increased risk of progression or death (Supplementary Figure S3).

Dichotomized with a threshold of 2 ng/mL, the high-risk subgroup comprised of 258 patients (37%) with a 3-year DFS of 66.4% (60.8-72.5), while the low-risk subgroup included 438 patients (63%) with a 3-year DFS of 80.9% (77.3-84.7; Figure 1B).

**Figure 2.**
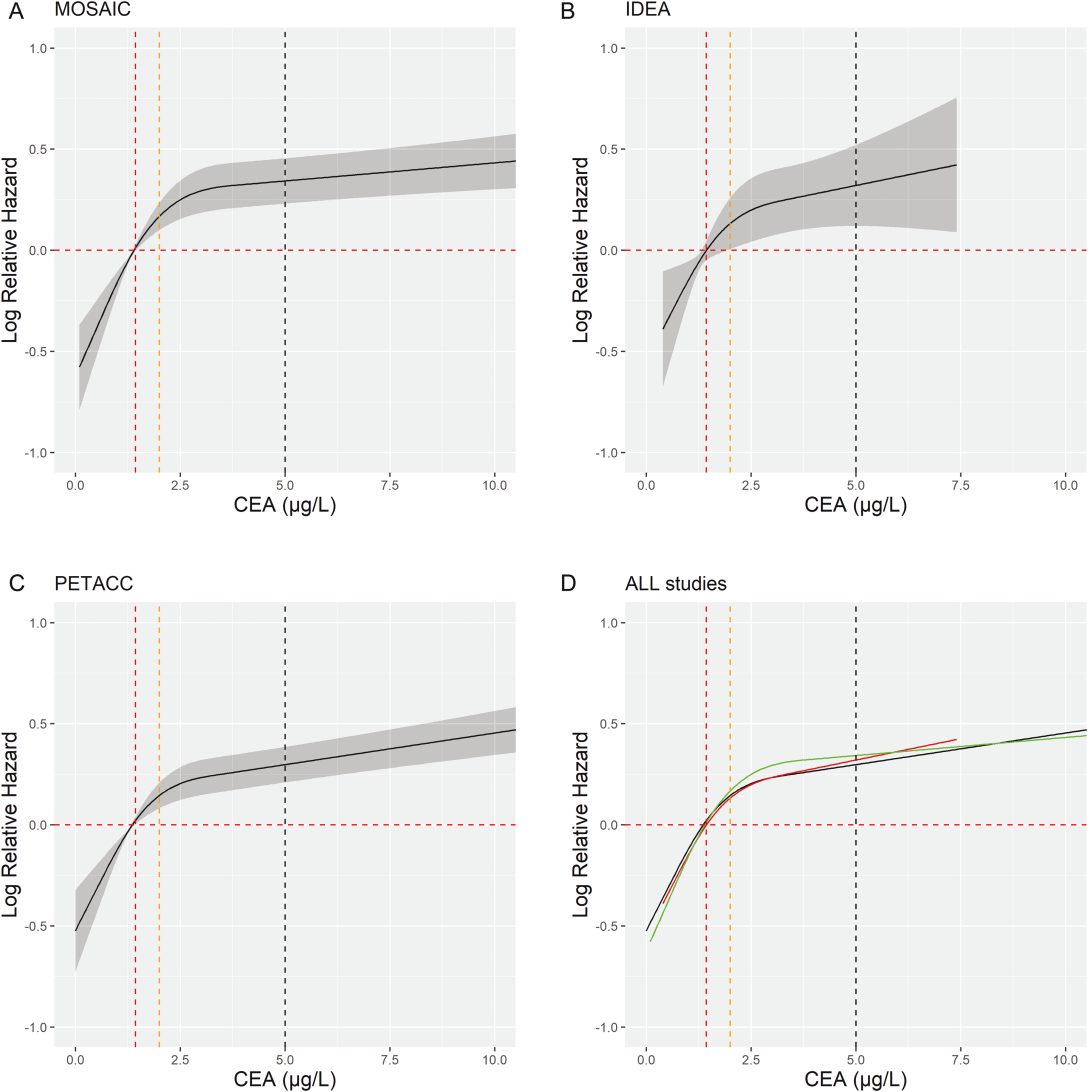
Association between CEA and DFS by the restricted cubic spline method for patients from (A) MOSAIC (*N* = 1292), (B) IDEA-France (*N* = 696), (C) PETTAC-08 (*N* = 2480), and (D) all trials to assess the reproducibility of CEA. Abbreviations: CEA, carcinoembryonic antigen; DFS, disease-free survival.

Reproducibility of this threshold was evaluated in 2 independent cohorts (MOSAIC, PETACC). The square-root relationship obtained by modeling DFS and CEA value was observed, with strikingly comparable results in all 3 cohorts (Figure 2).

### CEA, ctDNA, and pTN stage as independent prognostic factors for DFS

CEA value did not seem to correlate with ctDNA status. Among 146 patients with known ctDNA status and CEA ≥ 2 ng/mL, 127 patients (87%) were ctDNA negative. Similarly, out of 259 patients with known ctDNA status and CEA < 2 ng/mL, 26 (10%) were ctDNA positive (Supplementary Table S2).

In a multivariate Cox analysis adjusted for treatment duration, CEA ≥ 2 ng/mL (HR = 1.82, 95% CI: 1.27-2.59), ctDNA positivity (HR = 1.88, 95% CI:1.16-3.03), and pTN stage (HR = 1.78, 95% CI:1.24-2.54) were all identified as independent factors associated with DFS.

The relative contributions to the variability model were estimated through chi-square statistics, with CEA being the highest contributor. The respective chi-square values for CEA, pTN stage, and ctDNA were 10.5, 8.9, and 5.9 (Supplementary Figure S4).

### Combined analysis of CEA, ctDNA, and pTN stage

**Figure 3.**
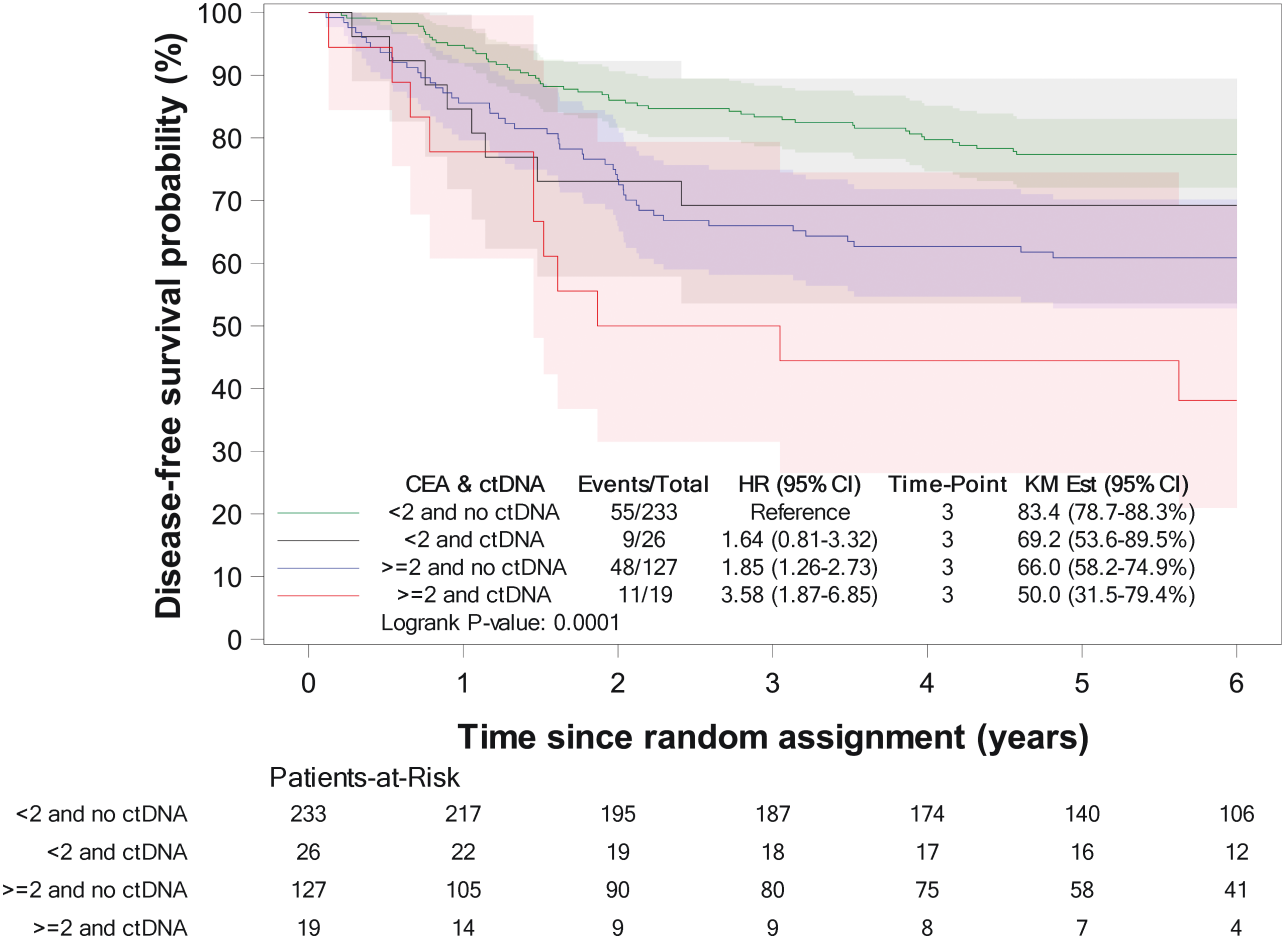
Kaplan-Meier estimates of DFS according to CEA and ctDNA status in patients with available CEA and ctDNA. Abbreviations: CEA, carcinoembryonic antigen; ctDNA, circulating DNA; DFS, disease-free survival.

In patients with both negative ctDNA status and CEA < 2 ng/mL, a high DFS was observed, with a Kaplan-Meier estimate at 3 years of 83.4% (78.7-88.3; Figure 3). If only one of these 2 risk factors was present, DFS decreased, with a 3-year DFS estimate of 69.2% (53.6-89.5) for ctDNA negative-only patients and of 66% (58.2-74.9) for CEA < 2 ng/mL-only patients. Patients with both positive ctDNA and CEA ≥ 2 ng/mL were at the highest risk of relapse, with a 3-year DFS estimate of 50% (31.5-79.4). Given very similar outcomes between the 2 subgroups of patients with only one factor absent, these were merged into a single intermediate-risk category, with a 3-year DFS estimate of 67% (59.4-74.6).

Further analyses showed that the pTN stage added discriminative value in patients with only one risk factor, but not in other cases (Supplementary Figure S5).

### New risk classification proposal

**Figure 4.**
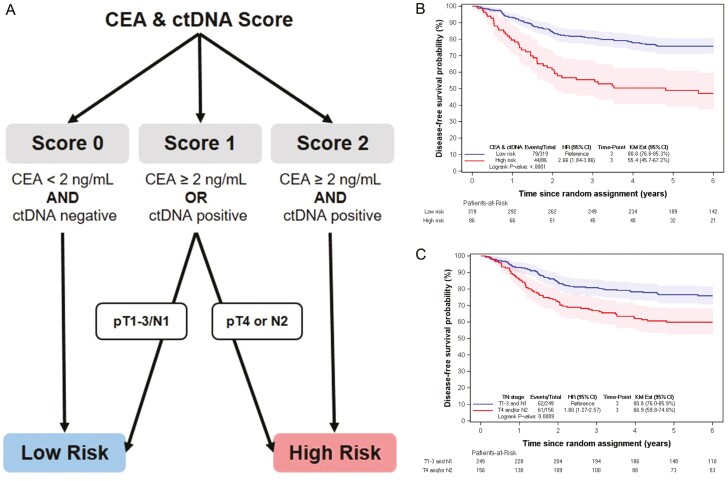
A new risk classification for stage 3 colorectal cancer (A). Kaplan-Meier estimates of DFS according to the new risk classification proposal (B), and according to the current classification based on pTN stage (C) in population with CEA and ctDNA. Abbreviations: CEA, carcinoembryonic antigen; DFS, disease-free survival.

A new classification is proposed to characterize the risk of relapse or death in stage 3 CC (Figure 4A).

In the first step, a combined biomarker score (CBS) is computed: if negative ctDNA and CEA ≤ 2 ng/mL, CBS = 0; if positive ctDNA with CEA ≤ 2 ng/mL OR negative ctDNA, with CEA ≥ 2 ng/mL, CBS = 1; if positive ctDNA and CEA ≥ 2 ng/mL, CBS = 2. Patients with CBS = 0 are classified as low-risk and those with CBS = 2 as high-risk. For patients with CBS = 1, those with pT1-3/N1 are classified as low-risk and with pT4/N2 as high-risk.

DFS estimates obtained with this new proposed risk classification were computed: 3-year DFS in low-risk was 80.8% (76.6-85.3) and in high-risk, it was 55.4% (45.7-67.2; HR = 2.66, 95% CI: 1.84-3.86, *P* < .001; Figure 4B). For comparison, pTN classification yielded a 3-year DFS of 80.8% (76.0-85.9) for low-risk patients (pT1-3/N1) and 66.9% (59.8-74.8) for high-risk patients (pT4/N2; HR = 1.80, 95% CI: 1.27-2.57, *P* < .001; Figure 4C).

The influence of each parameter in the new risk classification is shown in Table 2. Based on pTN-only classification, 249 (61%) patients would be considered low-risk and 156 (38%) as high-risk. In contrast, CBS-based classification would give a low-risk population of 319 patients (79%) and a high-risk population of 86 patients (21%). Notably, CBS enabled the reclassification of 80 patients with pT4/N2 to the low-risk group and the reassignment of 10 patients with pT3/N1 to the high-risk group. Sixty patients out of 86 in the CBS-based high-risk population had a CEA value between 2 and 5 ng/mL.

**Table 2.** Repartition of parameters of interest in the new risk classification.

	Final classification population		Low-risk group		High-risk group	
	*n* = 405		*n* = 319		*n* = 86	
	*n*	%	*n*	%	*n*	%
Tumor and node stage						
T1-3 and N1	249	61.5	239	74.9	10	11.6
T4 and/or N2	156	38.5	80	25.1	76	88.4
ctDNA						
Yes	45	11.1	17	5.3	28	32.6
No	360	88.9	302	94.7	58	67.4
CEA (ng/mL)						
<2	259	63.9	250	78.4	9	10.5
≥2 and <5	123	30.4	63	19.7	60	69.8
≥5	23	5.7	6	1.9	17	19.8

Abbreviations: CEA, carcinoembryonic antigen; ctDNA, circulating DNA.

### Predictive value of the new risk classification proposal for chemotherapy duration

The potential predictive value of this new classification for chemotherapy duration (6 vs 3 months mFOLFOX or CAPOX) was also investigated. In the cohort with evaluable CEA and ctDNA (*n* = 405), 89% of patients received mFOLFOX and 11% received CAPOX. Differences in DFS between 3-month and 6-month adjuvant chemotherapy according to the new classification are illustrated in Supplementary Figure S6 (interaction *P*-value = .078).

In the low-risk group (Supplementary Figure S6), a similar DFS was observed: the 3-year DFS rate was 78.8% for 3 months versus 82.8% for 6 months (HR = 1.23, 95% CI: 0.79-1.92; *P* = .35). In the high-risk group (Supplementary Figure S6), an improvement with 6 months of chemotherapy was observed: the 3-year DFS rate was 44.2% for 3 months and 67.5% for 6 months of treatment (HR, 2.38; 95% CI: 1.27-4.44, *P* = .005).

For comparison, pTN-only classification yielded an overall 3-year DFS rate of 79.7% versus 81.9% for 3 months versus 6 months of treatment in the pT1-3/N1 population (HR, 1.04, 95% CI:0.63-1.71, *P* = .88) and of 57.3% versus 76.3% in the pT4/N2 population (HR, 2.3995% CI:1.41-4.06, *P* < .001).

## Discussion

Standard clinical practice for adjuvant treatment in stage 3 colorectal cancer currently relies on pTN stage only. Our post hoc analysis of CEA values in the large phase 3 IDEA-France trial aimed to explore the potential for improved classification by integrating optimized CEA analysis and ctDNA status with the pTN stage. Previous studies based on the same population showed that ctDNA status alone is an independent prognostic marker, however with low predictive value for treatment duration, especially in pT1-3/N1 patients.^6^

An important aspect of this study was to evaluate the added value of CEA, emphasizing the significance of even slightly elevated CEA values. Mathematical modeling of DFS in this specific cohort led to the definition of an optimized threshold for CEA value at 2 ng/mL. It is essential to underscore that this determination was derived from a subset of patients, constituting 34% of the whole population (*n* = 696). Significantly, prior studies^10,11^ have also reported optimized cutoff values within a comparable range. For example, in the MOSAIC trial, a threshold of 2.35 ng/mL was pinpointed for stage 2 patients, while both the MOSAIC and PETTAC-8 trials demonstrated an optimized cutoff value of 1.30 ng/mL for stage 3 patients.

Combining both biomarkers with the pTN stage enabled us to define a new classification, expanding the low-risk group by almost one-third larger (319 patients vs 249) with a similar outcome (3-year DFS: 80.8% vs 80.8%). Conversely, the newly defined high-risk group included fewer patients but with a more severe outcome (3-year DFS: 55.4% vs 66.9%). Exploratory analysis of treatment duration suggested that this new classification also has predictive value, which was not evident with ctDNA status alone.^6^ To our knowledge, this is the first study proposing an integrated classification combining both CEA and ctDNA. Previous studies reported multivariate analyses showcasing the significance of postoperative CEA in predicting relapse. A Taiwanese study involving 141 patients with stage 2 and 3 cancers,^18^ identified postoperative CEA ≥ 5 ng/mL as the second most important prognostic factor (HR, 2.37) following persistent circulating tumor cells (HR, 11). Similarly, in a prospective study with 168 stage 3 patients,^12^ multivariate analysis of predictors for relapse yielded HR, 30.9 (10.6-90.2) for ctDNA, but postoperative CEA (with varying thresholds between countries and smoking status) remained a significant independent predictor alongside ctDNA, with HR, 3.43 (1.39-8.42). In a recent Japanese study of 1039 patients with stage 2-4 colorectal cancer, a discordance rate of 18.7% between CEA and ctDNA was reported.^8^ Moreover, the CEA value included in this study was measured at 12 weeks postsurgery, deviating from the standard 4-week interval. In the metastatic setting, both CEA and ctDNA have been identified as independent prognostic factors.^19^ It is worth highlighting that previous studies have relied on common CEA thresholds for abnormal values, a practice that probably diminishes its overall prognostic efficacy.

Altogether, these above-mentioned findings coupled with our comprehensive analysis of CEA and ctDNA status, suggest that these 2 biomarkers are not redundant. Instead, they provide complementary information that enhances our ability to better characterize patients at higher risk of relapse.

Limitations of our study include unplanned ctDNA analysis with suboptimal preanalytical conditions and the use of tumor-agnostic digital droplet methylation status,^17^ distinct from personalized blood analysis derived from patient biopsy used in other studies. Performance between these methods has not been directly compared. The ctDNA positivity rate reported here was relatively low (13.8%), in the range of values previously reported (between 10% and 90%).^17,20-22^

Recent work suggests that longitudinal analyses of ctDNA may further improve its prognostic performance.^23^ Serial ctDNA was not available in IDEA-France and could therefore not be evaluated. It should however be noted that longitudinal analyses also add complexity for guiding adjuvant treatment. For instance, it would seem difficult to start chemotherapy in cases of patients whose ctDNA becomes positive a few months after surgery.

Another limitation arises from the small subset of the initial population in the IDEA-France trial, which could be included in our analysis. The retrieval of CEA values was not exhaustive, as they were not systematically recorded in the medical files of all patients. Additionally, the ctDNA status was missing for some patients. However, it is noteworthy that combined data (encompassing both ctDNA and CEA value), were available for 405 patients. Although we could not eliminate the possibility of selection bias, the main characteristics and prognosis of patients with or without CEA values or ctDNA status were comparable. The prognostic and predictive values of the proposed classification need however to be validated in an external cohort. Finally, the analysis of treatment duration with respect to the new classification was impaired by the fact that most patients in our cohort (90%) received mFOLFOX6. Previous data have shown an interaction between the type of regimen used and the duration of chemotherapy. Consequently, the impact of the new classification with the CAPOX regimen remains thus unknown and warrants further investigation.

## Conclusion

Our results underscore the importance of a combined analysis of biological, molecular, and histological markers to tailor treatment for each patient with stage 3 CC. Systematic recording of CEA values along ctDNA status should be reported along traditional risk factors in further studies, potentially reshaping the landscape of risk stratification and treatment decisions in stage 3 CC.

## Supplementary material

Supplementary material is available at *The Oncologist* online.

oyae140_suppl_Supplementary_Material

## Data Availability

Data are available from the corresponding author upon reasonable request.
