# Supplementary material for: A novel risk classification model integrating CEA, ctDNA, and pTN stage for stage 3 colon cancer: a post hoc analysis of the IDEA-France trial
Source: Oncologist. 2024 Jul 15;29(11):e1492–500. doi: 10.1093/oncolo/oyae140 (PMC11546770; doi:10.1093/oncolo/oyae140)
Supplement: oyae140_suppl_Supplementary_Material [file oyae140_suppl_supplementary_material.docx]

##

**Supplementary Figure S1 –** Flowchart

291 patients with missing ctDNA status

1314 patients with missing CEA values

IDEA-France mITT population N = 2010

CEA value available n = 696

CEA value and ctDNA status available n = 405

mIIT, modified intent-to-treat population; CEA, carcinoembryonic antigen; ctDNA, circulating DNA

## **Supplementary Figure S2 –** Kaplan-Meier estimates of DFS in patients with or without CEA


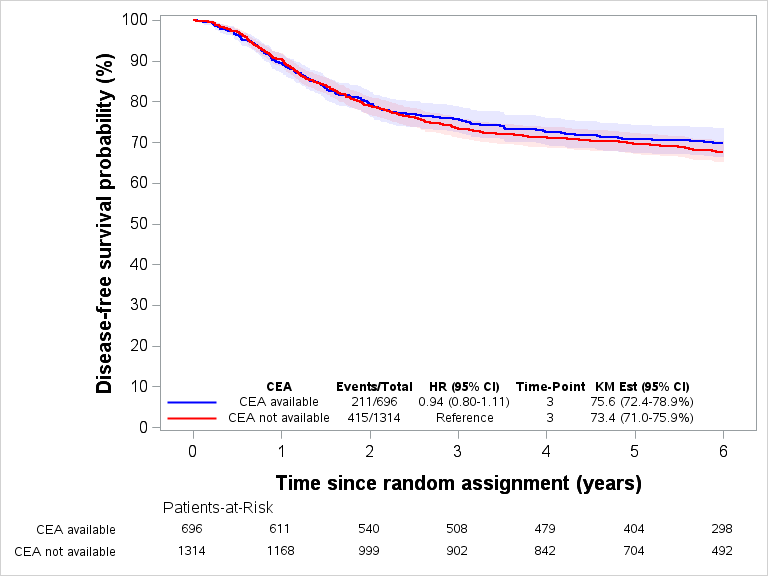


CEA, carcinoembryonic antigen; DFS, disease-free survival

**Supplementary Figure S3 -** Kaplan-Meier estimates of DFS according to CEA values. Binary classification with common 5 ng/mL threshold (A). 3-group classification for CEA <2 ng/mL, ≥2 and ≥5 ng/mL, and ≥5 ng/mL (B). Binary classification with optimized 2 ng/mL threshold (C).

| A  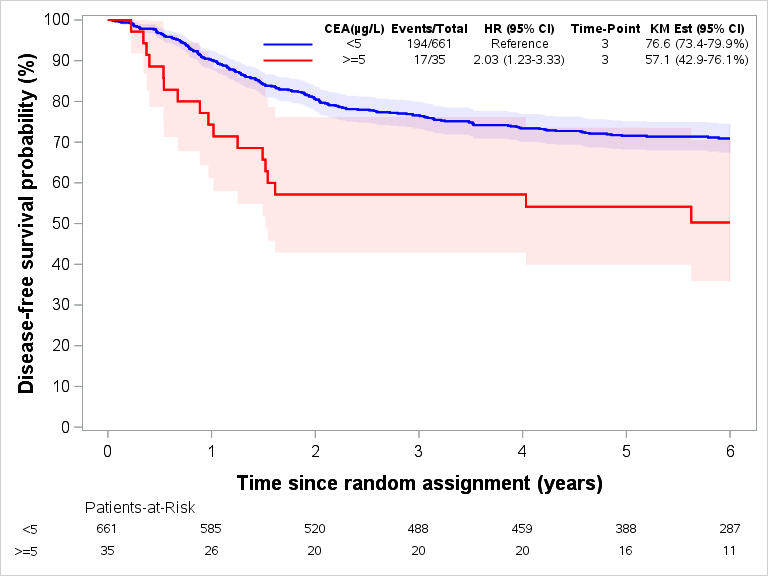 | B  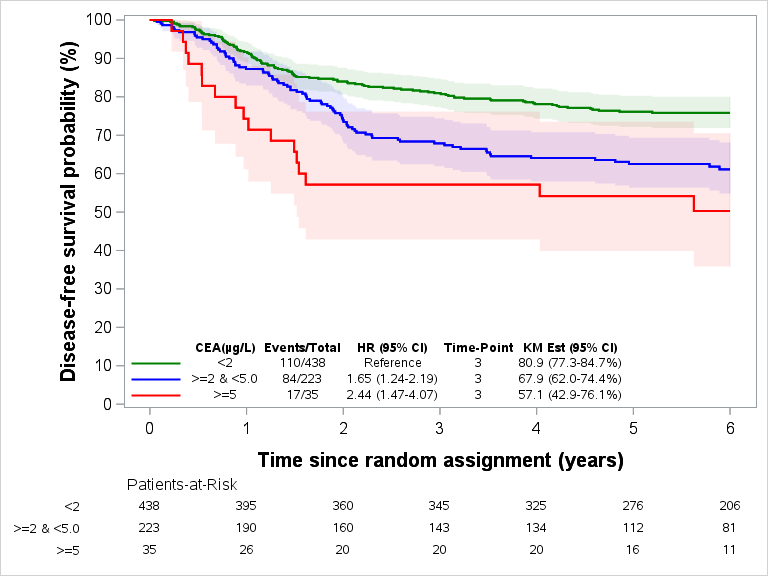 | C  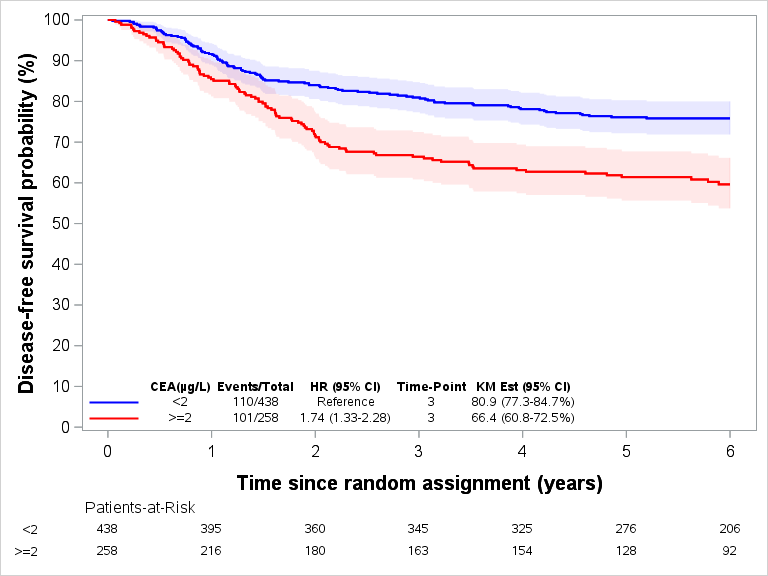 |
| --- | --- | --- |

CEA, carcinoembryonic antigen; DFS, disease-free survival

**Supplementary Figure S4 –** Chi2 value associated with each variable in multivariate Cox model analysis


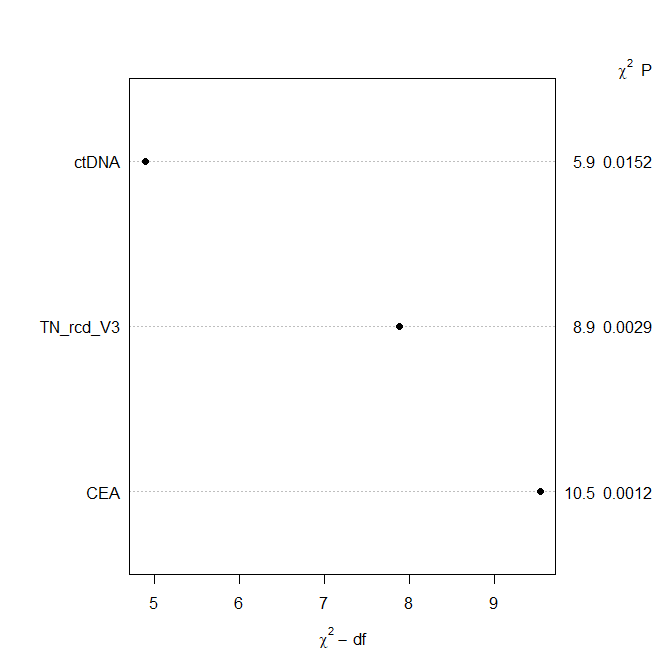


CEA, carcinoembryonic antigen; ctDNA, circulating DNA; TN, tumor node

**Supplementary Figure S5 –** Kaplan-Meier estimates of DFS according to CBS in patients with pT1-3/N1 (A) and pT4/N2 (B)

| A  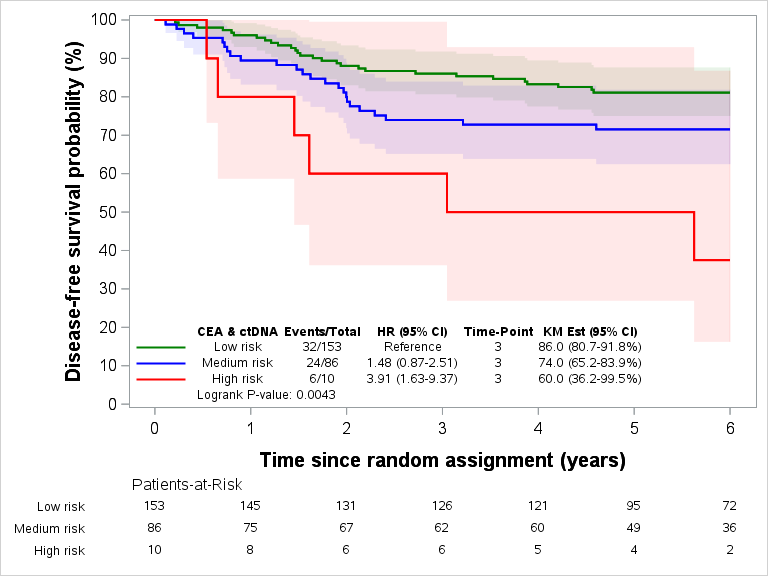 | B  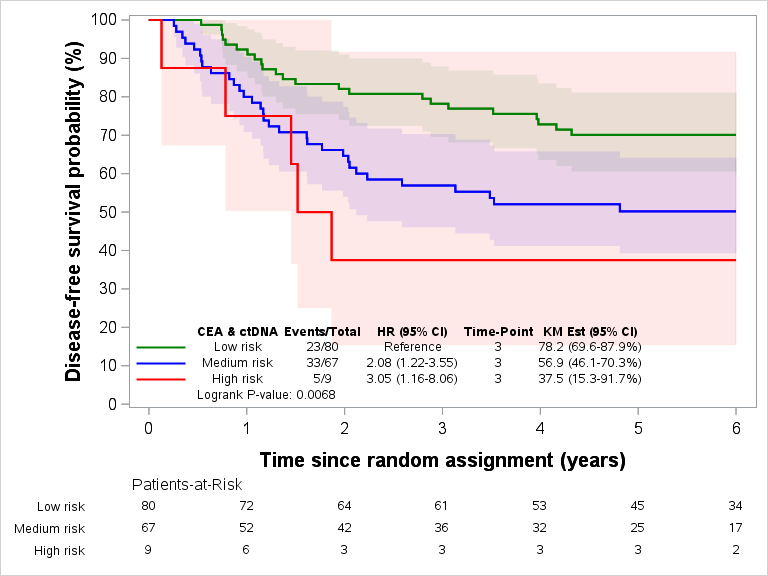 |
| --- | --- |

CEA, carcinoembryonic antigen; ctDNA, circulating DNA; DFS, disease-free survival; CBS, combined biomarker score

**Supplementary Figure S6 -** Kaplan-Meier estimates of DFS according to the duration of treatment (6 months versus 3 months) in patients with low-risk (A) and high-risk (B) by the new risk classification proposal

| A  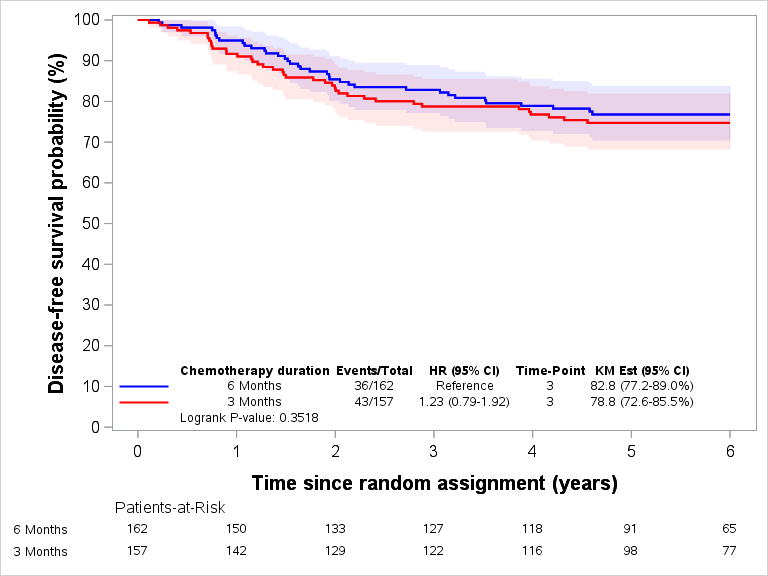 | B  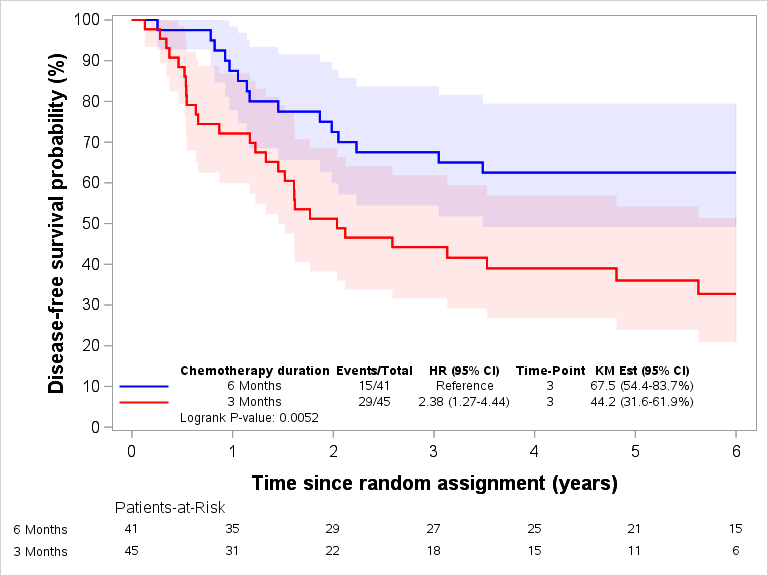 |
| --- | --- |

DFS, disease-free survival

**Supplementary Figure S7 -** Kaplan-Meier estimates of DFS with common non-optimized CEA threshold (5 ng/mL). Combined analysis with CEA and ctDNA (A) and integrated classification with CEA, ctDNA, and pTN (B)

| A  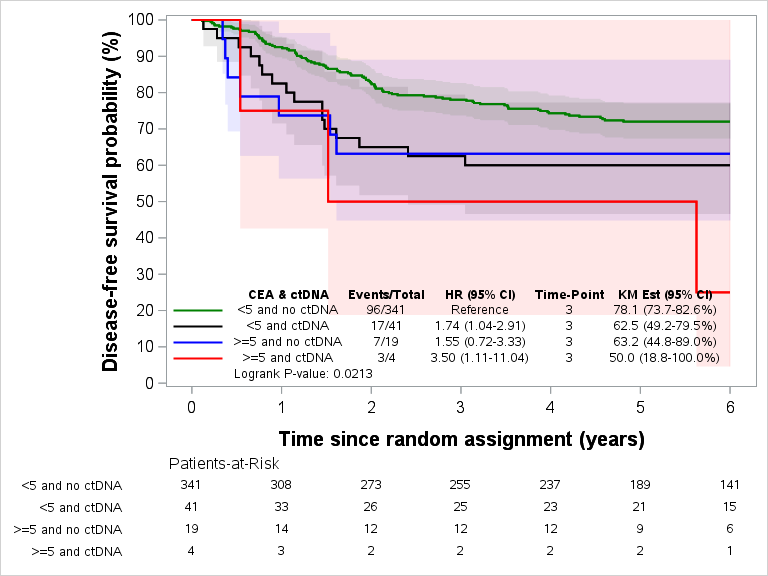 | B  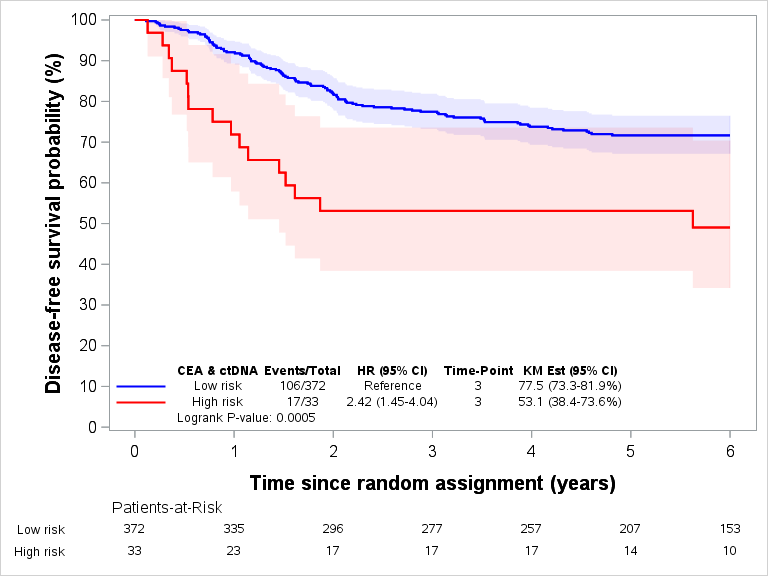 |
| --- | --- |

**Supplementary Table S1 –** Characteristics of patients with or without available CEA

|  | **mITT population** | | **Population with CEA** | | **Population without CEA** | |
| --- | --- | --- | --- | --- | --- | --- |
|  | n = 2010 | | n = 696 | | n = 1314 | |
|  | n | % | n | % | n | % |
| **Age (years)** |  |  |  |  |  |  |
| Mean (sd) | 63.9 (9.4) | | 62.8 (9.6) | | 64.5 (9.2) | |
| Median (Q1-Q3) | 64.7 (58.1-70.8) | | 63.8 (57.1-69.3) | | 65.4 (58.7-71.5) | |
| ≤70 | 1443 | 71.8 | 533 | 76.6 | 910 | 69.3 |
| >70 | 567 | 28.2 | 163 | 23.4 | 404 | 30.8 |
| **Sex** |  |  |  |  |  |  |
| Male | 1144 | 56.9 | 373 | 53.6 | 771 | 58.7 |
| Female | 866 | 43.1 | 323 | 46.4 | 543 | 41.3 |
| **ECOG PS** |  |  |  |  |  |  |
| 0 | 1479 | 73.6 | 533 | 76.6 | 946 | 72.0 |
| 1-2 | 531 | 26.4 | 163 | 23.4 | 368 | 28.0 |
| **Tumor and node stage** |  |  |  |  |  |  |
| T1-3 and N1 | 1246 | 62.0 | 430 | 61.8 | 816 | 62.1 |
| T4 and/or N2 | 764 | 38.0 | 266 | 38.2 | 498 | 37.9 |
| **Chemotherapy duration** |  |  |  |  |  |  |
| 3 months  FOLFOX  CAPOX | 1002  895  107 | 49.9  89.3  10.7 | 347  298  49 | 49.9  85.9  14.1 | 655  597  58 | 49.9  91.1  8.9 |
| 6 months  FOLFOX  CAPOX | 1008  914  94 | 50.1  90.7  9.3 | 349  306  43 | 50.1  87.7  12.3 | 659  608  52 | 50.1  92.3  7.7 |
| **Primary tumor site** |  |  |  |  |  |  |
| Left | 1161 | 60.4 | 435 | 63.4 | 726 | 58.7 |
| Right | 746 | 38.8 | 246 | 35.9 | 500 | 40.4 |
| Both | 16 | 0.8 | 5 | 0.7 | 11 | 0.9 |
| Missing | 87 | - | 10 | - | 77 | - |
| **Histologic grade** |  |  |  |  |  |  |
| Low grade | 1764 | 91.7 | 592 | 89.8 | 1172 | 92.7 |
| High grade | 159 | 8.3 | 67 | 10.2 | 92 | 7.3 |
| Missing | 87 | - | 37 | - | 50 | - |
| **ctDNA** |  |  |  |  |  |  |
| Yes | 140 | 13.8 | 45 | 11.1 | 95 | 15.6 |
| No | 875 | 86.2 | 360 | 88.9 | 515 | 84.4 |
| Missing | 995 | - | 291 | - | 704 | - |

mIIT, modified intent-to-treat population; CEA, carcinoembryonic antigen; ctDNA, circulating DNA; ECOG PS, Eastern Cooperative Oncology Group Performance Status

**Supplementary Table S2 -** Patient characteristics according to CEA value

| **Characteristics** | **Population with CEA** | | **CEA <2** | | **CEA ≥2** | |
| --- | --- | --- | --- | --- | --- | --- |
|  | n = 696 | | n = 438 | | n = 258 | |
|  | n | % | n | % | n | % |
| **Age, years** |  |  |  |  |  |  |
| Mean (sd) | 62.8 (9.6) | | 62.3 (9.9) | | 63.6 (9.2) | |
| Median (Q1-Q3) | 63.8 (57.1-69.3) | | 63.4 (56.3-69.1) | | 64.4 (58.3-70.3) | |
| ≤70 | 533 | 76.6 | 345 | 78.8 | 188 | 72.9 |
| >70 | 163 | 23.4 | 93 | 21.2 | 70 | 27.1 |
| **Sex** |  |  |  |  |  |  |
| Male | 373 | 53.6 | 227 | 51.8 | 146 | 56.6 |
| Female | 323 | 46.4 | 211 | 48.2 | 112 | 43.4 |
| **ECOG PS** |  |  |  |  |  |  |
| 0 | 533 | 76.6 | 330 | 75.3 | 203 | 78.7 |
| 1-2 | 163 | 23.4 | 108 | 24.7 | 55 | 21.3 |
| **Tumor and node stage** |  |  |  |  |  |  |
| T1-3 and N1 | 430 | 61.8 | 284 | 64.8 | 146 | 56.6 |
| T4 and/or N2 | 266 | 38.2 | 154 | 35.2 | 112 | 43.4 |
| **Chemotherapy duration** |  |  |  |  |  |  |
| 3 months  FOLFOX  CAPOX | 347  298  49 | 49.9  85.9  14.1 | 214  186  28 | 48.9  86.9  13.1 | 133  112  21 | 51.6  84.2  15.8 |
| 6 months  FOLFOX  CAPOX | 349  306  43 | 50.1  87.7  12.3 | 224  198  26 | 51.1  88.4  11.6 | 125  108  17 | 48.4  86.4  13.6 |
| **Primary tumor site** |  |  |  |  |  |  |
| Left | 435 | 63.4 | 286 | 66.2 | 149 | 58.7 |
| Right | 246 | 35.9 | 141 | 32.6 | 105 | 41.3 |
| Both | 5 | 0.7 | 5 | 1.2 | 0 | 0.0 |
| Missing | 10 | - | 6 | - | 4 | - |
| **Histologic grade** |  |  |  |  |  |  |
| Low grade | 592 | 89.8 | 374 | 90.6 | 218 | 88.6 |
| High grade | 67 | 10.2 | 39 | 9.4 | 28 | 11.4 |
| Missing | 37 | - | 25 | - |  |  |
| **ctDNA** |  |  |  |  |  |  |
| Yes | 45 | 11.1 | 26 | 10.0 | 19 | 13.0 |
| No | 360 | 88.9 | 233 | 90.0 | 127 | 87.0 |
| Missing | 291 | - | 179 | - | 112 | - |

mIIT, modified intent-to-treat population; CEA, carcinoembryonic antigen; ctDNA, circulating DNA; ECOG PS, Eastern Cooperative Oncology Group Performance Status
